# External Radiation and Brachytherapy Resource Deficit for Cervical Cancer in India: Call to Action for Treatment of All

**DOI:** 10.1200/JGO.18.00250

**Published:** 2019-06-05

**Authors:** Supriya Chopra, Richa Shukla, Atul Budukh, Shyam Kishore Shrivastava

**Affiliations:** ^1^ Tata Memorial Centre, Homi Bhabha National Institute, Navi Mumbai, India; ^2^ Apollo Hospital, Navi Mumbai, India

## Introduction

Cervical cancer is the fourth most common cancer among women worldwide, with India contributing to 17% of the new cases. Overall, more than 96,000 new cases are diagnosed in India per year, with average age standardized rates of incidence and mortality of 14.9 and 9.2 per 100,000, respectively.^1-3^ The high mortality-to-incidence ratio^4^ suggests not only that patients are diagnosed in advanced stages, but that they may also have delayed or suboptimal access to treatment. Optimal chemoradiation and brachytherapy (BT) in locally advanced cervical cancer (LACC) are associated with 5-year survival rates of 75% and 55% in those with stage IB2 to IIB and IIIB disease, respectively, in clinical trials and tertiary care centers in India.^5-8^ Therefore, the National Cancer Grid of India and Indian Council of Medical Research recommend that chemoradiation and BT be the standard of care for LACC.^9,10^

Because most women with cervical cancer in India (as well as in other low-income countries) present with locally advanced–stage disease, the overall need (in both adjuvant and definitive settings) is expected to be as high as 85% to 90%.^7,8,11,12^ However, it is unclear if all women with cervical cancer have access to external-beam radiotherapy (EBRT; also known as teletherapy) or BT. A recent analysis reported that overall, only 35% to 40% of patients in India might have access to RT; however, no specific details are available for cervical cancer.^13^ A report from India that detailed state-level EBRT and BT infrastructure in 2008 focused on providing projections for EBRT infrastructure only.^14^ Similarly, international RT access initiatives focus essentially on EBRT access.^15-17^ Although therapeutic needs in most cancers can be projected by estimating EBRT deficit, this is not the case for cervical cancer, because paired availability of EBRT and BT is crucial for cure. Also, because there is substantial variation in incidence of cervical cancer across Indian states (5.6 to 24.3 per 100,000),^18^ it is important that infrastructural needs are projected in reference to state-level rather than national incidence.

We therefore undertook a study to report access to EBRT and BT treatment units in reference to state-level incidence of cervical cancer and calculate the unmet infrastructural needs. This study was planned with the aim of providing a guidance document for development of RT infrastructure for cervical cancer in India.

## Methods

The number of cervical cancer cases was estimated for each state and union territory using the nearest population-based cancer registry and verified against published data of overall incidence, including the Globocan 2018 report.^2,3,18^ State-level availability of EBRT and BT resources was obtained through available RT facility databases. The absolute number of cervical cancer cases in each state was obtained by multiplying the age-specific incidence rates with the respective age subgroups of the female population, and an incidence map was generated. A rate of RT use of 85% was estimated based on available data on incidence of cervical cancer according to stage.^7,8^ External radiation fractions needed were calculated on the basis of 25 common fraction schedules as follows: total number of fractions needed for each state = cervical cancers in the state × 0.85 × 25. Presuming that patients with LACC will need four fractions of BT, the overall BT fractions needed were estimated as: cervical cancer cases in state × 0.85 × 4. On the basis of proportionate incidence of cervical cancer and use of external RT machine space across various departments, it was presumed that 10% of the available EBRT infrastructural resources would be allocated for treatment of cervical cancer.^2^ For available BT machines, 100% use was presumed for cervical cancer.

Total EBRT treatment capacity for cervical cancer (in number of fractions) was estimated by multiplying the number of units per state with 240 working days (assuming 5 days per week and accounting for annual holidays). Assuming that 50 patients would be treated per day, a proportional treatment space of 10% was estimated for cervical cancer. Therefore, feasible fractions were estimated as: number of EBRT units per state × 240 × 0.10 × 50. Similarly, BT capacity was estimated at four procedures per unit per day for 240 days. BT fraction capacity was therefore calculated as: number of BT units × 240 × 4. Deficit in EBRT and BT fractions and subsequently number of EBRT and BT units were calculated by the difference between available and needed infrastructure.

**CONTEXT**

**Key Objective**
The study maps availability of radiotherapy (RT) treatment units regarding state-level incidence and need for RT in cervical cancer in India.
**Knowledge Generated**
Results show the need for 109 external RT units (or 10% of space in 1,090 required external-beam RT units) and 127 brachytherapy (BT) units to meet the treatment needs for cervical cancer. If resource-sharing models are developed between states, an additional 58 BT units will still be needed. With the current resources, close to 14,000 women with cervical cancer will have delayed access to treatment.
**Relevance**
Results of our study can serve as guidance for financial investors and health policymakers.

## Results

**FIG 1:**
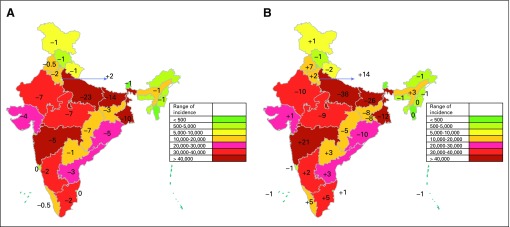
(A) External-beam radiotherapy (EBRT) and (B) brachytherapy deficits in state-level incidence of cervical cancer in India. Assumes 10% machine space for patients with cervical cancer in EBRT units.

Table 1 and Figures 1A and 1B summarize the state-level deficit of EBRT and BT equipment for treating cervical cancer. Overall, 22 Indian states had a deficit in equipment for EBRT, with additional needs of 1 to 24 units just for treating cervical cancer. A total of 14 states had a deficit in BT units (1 to 38 units). If state-level deficit is taken into account, an additional 109 EBRT units (or 10% of the space of 1,090 additional EBRT units) and 127 BT units are needed just for patients with cervical cancer.

**TABLE 1:**
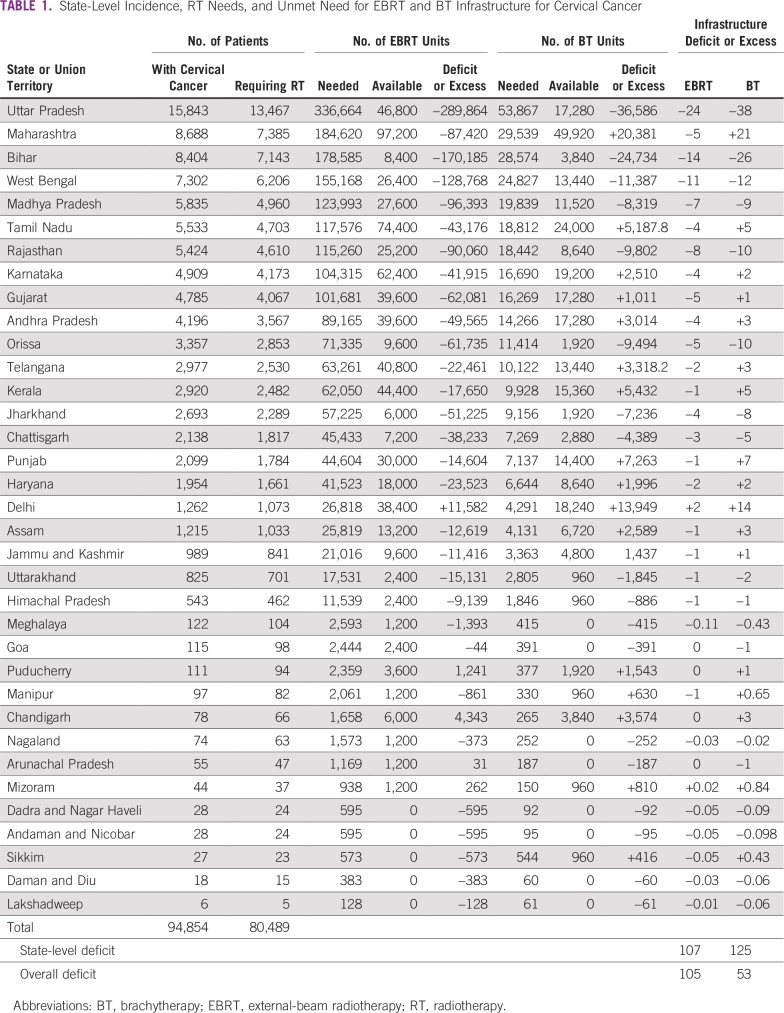
State-Level Incidence, RT Needs, and Unmet Need for EBRT and BT Infrastructure for Cervical Cancer

Infrastructural deficit for cervical cancer was most pronounced in the high-incidence states of Uttar Pradesh, Bihar, West Bengal, Odisha, and Rajasthan, suggesting the potential for a serious survival disadvantage after diagnosis of cervical cancer in these states. It was observed that although no states had excess EBRT resources, few states had excess BT treatment units in reference to baseline incidence of cervical cancer. If the excess treatment units of adjacent states are taken into account, an additional 58 BT units will still be needed. It is estimated that with the current BT deficit, approximately 14,000 women in India may have delayed or no access to BT, thereby precluding cure.

## Discussion

Cervical cancer mortality represents a great threat to women’s health, with one death every 2 minutes estimated worldwide. The WHO has issued a call to action for the elimination of cervical cancer, with the main emphasis on vaccination, screening, and treatment of early lesions and palliative care and limited emphasis on RT availability.^19^ Although vaccination and screening are likely to have an impact on incidence and mortality reduction over the next few decades,^20^ access to RT resources will be crucial for the treatment of a majority of patients with cervical cancer.

To our knowledge, our report presents the first state-level incidence-based evaluation of RT resources for the treatment of LACC in India. Our results highlight the regional disparity in EBRT and BT treatment unit allocation and overall deficit of treatment units for treating LACC. Our analysis also reveals geographic clustering of therapeutic units. Although resource-sharing models between different states for EBRT and BT may seem to be a potential interim solution, the implementation of such may be difficult in India because of challenges related to the need to travel long distances and find interim housing, inadequate financial resources, out-of-pocket expenditures, and inadequate medical insurance coverage.^21,22^ Increasing travel distance to receive care is known to be associated with reduced rates of treatment completion and has had adverse effects on survival in patients with cervical cancer^23,24^ in underserved regions in the United States; this is likely to be the same in India and other developing countries. Results from ongoing and completed resource-sparing BT trials are awaited to understand if existing resources can be used more optimally.^25,26^ Furthermore, resource sharing between geographically close institutions or institutional networks (like the National Cancer Grid of India)^9^ may help bridge the deficit in treatment units, and such partnership models should be prospectively investigated.

Whereas global initiatives exist to improve access to EBRT,^15-17^ there are no structured international initiatives to map BT resources, which is critical to cure of cervical cancer. Therefore, a formal global assessment of treatment units and practices must be undertaken. It is noteworthy that the resource deficit for cervical cancer is reported not only in low- to middle-income countries but in high-income countries as well. Multiple studies have reported suboptimal use of cervical BT, including access to facilities, increased overall treatment time, and sometimes omission of BT in favor of less effective external RT techniques.^27-29^ A need for a call to international action involving multiple stakeholders was therefore recently discussed at the World Cancer Congress,^30^ and a joint international initiative is being envisaged to map international access to cervical cancer EBRT and BT. It is also predicted that the joint initiative would work toward a financial investment plan and estimate the impact of inaction, with an aim of improving international infrastructure for treatment of LACC in the next decade. Although we report on a treatment unit deficit and the potential for undertreatment in reference to incidence, our work relies on assumptions related to number of fractions of EBRT and BT and human resources. A national and international systematic survey related to real practice, available equipment (eg, applicators, imaging units, and human resources) would be needed to further strengthen the estimates of resource deficit.

In conclusion, access to EBRT (and concurrent chemotherapy) and BT is crucial for achieving local control and improving outcomes of patients with locally advanced cervical cancer. There is a clear need to estimate worldwide resources to ensure treatment of all until eradication becomes a reality.
